# Transition in care from paramedics to emergency department nurses: a systematic review protocol

**DOI:** 10.1186/s13643-017-0651-z

**Published:** 2017-12-19

**Authors:** Gudrun Reay, Jill M. Norris, K. Alix Hayden, Joanna Abraham, Katherine Yokom, Lorelli Nowell, Gerald C. Lazarenko, Eddy S. Lang

**Affiliations:** 1 Faculty of Nursing, University of Calgary, Calgary, Canada; 2 Libraries and Cultural Resources, University of Calgary, Calgary, Canada; 3 Department of Biomedical and Health Information Sciences, College of Applied Health Sciences,, University of Illinois, Champaign, USA; 4 Emergency Medical Services, Calgary Zone, Alberta Health Services, Alberta Health Services, Calgary, Canada; 5 Taylor Institute for Teaching and Learning, University of Calgary, Calgary, Canada; 6 Department of Emergency Medicine, Cumming School of Medicine, University of Calgary, Calgary, Canada; 7 Pharmacy Services, Alberta Health Services, Edmonton, Canada; 8 Alberta Health Services, Emergency Medicine, Calgary Zone, Calgary, Canada

**Keywords:** Systematic review, Handover, Transfer of care, Transition in care, Emergency department triage, Emergency medical services, Paramedics

## Abstract

**Background:**

Effective and efficient transitions in care between emergency medical services (EMS) practitioners and emergency department (ED) nurses is vital as poor clinical transitions in care may place patients at increased risk for adverse events such as delay in treatment for time sensitive conditions (e.g., myocardial infarction) or worsening of status (e.g., sepsis). Such transitions in care are complex and prone to communication errors primarily caused by misunderstanding related to divergent professional perspectives leading to misunderstandings that are further susceptible to contextual factors and divergent professional lenses. In this systematic review, we aim to examine (1) factors that mitigate or improve transitions in care specifically from EMS practitioners to ED nurses, and (2) effectiveness of interventional strategies that lead to improvements in communication and fewer adverse events.

**Methods:**

We will search electronic databases (DARE, MEDLINE, EMBASE, Cochrane, CINAHL, Joanna Briggs Institute EBP; Communication Abstracts); gray literature (gray literature databases, organization websites, querying experts in emergency medicine); and reference lists and conduct forward citation searches of included studies. All English-language primary studies will be eligible for inclusion if the study includes (1) EMS practitioners or ED nurses involved in transitions for arriving EMS patients; and (2) an intervention to improve transitions in care or description of factors that influence transitions in care (barriers/facilitators, perceptions, experiences, quality of information exchange). Two reviewers will independently screen titles/abstracts and full texts for inclusion and methodological quality. We will use narrative and thematic synthesis to integrate and explore relationships within the data. Should the data permit, a meta-analysis will be conducted.

**Discussion:**

This systematic review will help identify factors that influence communication between EMS and ED nurses during transitions in care, and identify interventional strategies that lead to improved communication and decrease in adverse events. The findings can be used to develop an evidence-informed transitions in care tool that ensures efficient transfer of accurate patient information, continuity of care, enhances patient safety, and avoids duplication of services. This review will also identify gaps in the existing literature to inform future research efforts.

**Trial registration:**

PROSPERO CRD42017068844

## Background

Transitions in care between healthcare providers are complex processes that can result in vital information being lost or altered [1–3]. Three points of transition in care can occur for patients arriving in the Emergency Department (ED) via Emergency Medical Services (EMS): (1) EMS to triage nurse, (2) EMS to bedside nurse, and (3) EMS to ED trauma team. Efficient and safe transitions in care are dependent on accurate communication of pertinent patient information between health service providers [4, 5]. Information transfer during transitions between EMS-ED nurses can be hampered by multiple factors, including interruptions, multitasking, workload, and suboptimal working relationships [2, 6, 7]. This can result in loss of key information [6], which may negatively impact patient safety. Poor clinical transitions in care within the ED between care providers may place patients at increased risk for harm, such as overlooking early deterioration, risk of transferring cognitive bias, and delayed or missed investigations [5, 8, 9]. It is, thus, vital that transitions in care within and between professional groups providing ED care, including EMS practitioners-ED nurses, is accurate and efficient.

EMS practitioners and ED nurses have different professional perspectives. EMS practitioners in the pre-hospital setting focus on information that enables them to determine underlying pathology/injury, required treatments, and destination [10–12]. They have key insights about the unfolding of events, goals of care, and the patient’s environment. Furthermore, they are aware that waits at the hospital result in fewer ambulances being available to respond to emergency calls. ED triage nurses, in addition to determining acuity and prioritizing patients, are responsible for appropriate allocation of treatment space in the ED [13–15]. Hence, they require information that will enable them to predict the trajectory of patients and resources (staff and equipment) required.

Loss of information during transitions in care may lead to incorrect triage decisions in terms of acuity rating or prioritization of order to be examined by a physician, delayed time to treatment, or the patient being triaged to an inappropriate area of the ED [16, 17]. Ultimately, this impacts patient safety, patient flow, and the efficient and timely use of ED resources. Triage decisions determine a patient’s destination in the ED; therefore, the patient’s trajectory through the ED may be influenced by decisions made at triage. For instance, a patient who is erroneously triaged to a less acute area in the ED may be perceived as less urgent by the treating physician [16]. An effective triage system in which patients are accurately triaged is thus essential for the safety of patients who present to the ED, for managing patient flow through the ED, and for the appropriate use of ED resources [18–21].

Preliminary findings from two studies conducted by GR—one of decision-making by paramedics (in progress) and one of decision-making by ED triage nurses—indicate that EMS-triage nurse communication is problematic [15]. Paramedics expressed that triage nurses did not always trust them, that their professional knowledge and clinical expertise was at times disregarded, and that nurses did not adequately consider the patient information paramedics reported. Nurses did not fully trust information from EMS and, at times, felt they were given borderline misleading information in order for paramedics to be triaged faster [22]. Similar findings have been reported by others [23]. Mistrust was not always the case: some participants spoke of “good” paramedics and “good” triage nurses with whom they had an excellent working relationship. The outcome of mistrust is the potential for loss of vital patient information during the transition process at triage and at the bedside.

A related UK-based literature review [1] identified similar concerns about interprofessional relationships, particularly mistrust and misunderstandings between prehospital and hospital staff during transitions in care. Communication and information transfer were also susceptible to issues with context and environment. The review, however, was methodologically weak (e.g., risk of bias assessment and study characteristics are not detailed, limited dates and searches, no gray literature searches, focus on UK). Furthermore, this review did not focus on the factors that can improve the quality of transitions in care or evaluate the effectiveness of interventions to support transitions of care between EMS and ED nurses. To address these limitations and ensure that healthcare policy-makers have high quality evidence from which to make accurate decisions, we will conduct a comprehensive systematic review of studies that examine transitions in care between EMS practitioners and ED nurses.

### Aim

The objectives of this systematic review are to assess factors that mitigate or improve transitions in care from EMS practitioners to ED nurses and to identify effective interventional strategies that lead to improvements in communication and a reduction in adverse events.

## Methods

### Design

Our review question goes beyond *what works* to understand the perceptions, attitudes, and beliefs of those directly involved in transitions in care. Based on a preliminary scoping search, we expect diverse observational and qualitative studies of transitions in care. As such, we will conduct a mixed systematic review (i.e., mixed studies or mixed methods review) [24, 25] by combining the findings of diverse primary studies within a single review to address overlapping review questions. This protocol adheres to the PRISMA-P statement [26] and has been registered with PROSPERO CRD42017068844.

### Eligibility criteria

The questions of relevance are as follows:

1. What factors mitigate or improve transitions in care from EMS practitioners to ED nurses?
2. What interventional strategies lead to improvements in communication and a reduction in adverse events?

#### Participants

Studies will be eligible if they include EMS practitioners (paramedics, emergency medical technicians, registered nurses, licensed practical nurses, nurse practitioners) and/or ED nurses (registered nurses, licensed practical nurses, nurse practitioners) involved in transitions in care of EMS patients from the ambulance to the ED. Other modes of transfer can occur (e.g., from accompanying transfer nurse or physician); however, the aim of our review is to specifically examine transition in care form EMS practitioners to ED nurses as this is the most common route. If the study participants include other healthcare providers (e.g., physicians, allied health, unit clerk), we will include the study only if data for EMS practitioners and/or ED nurses are reported separately. We will exclude studies that report solely on transitions in care between EMS practitioners and the ED trauma team, physicians, allied health professionals, and unit clerks. Studies in which transitions in care occurs from police, peace officers, referring physicians, referring nurses, referring allied health professionals to triage nurses will also be excluded.

#### Interventions

If the study includes an intervention, it must focus on improving transitions in care from EMS to triage nurses or bedside nurses within hospital EDs, to be included. Studies that do not include an intervention are also eligible. Interventions may include, but will not be limited to standardized protocols for handover, checklists, mnemonics, automatic electronic data transfer, or communication skills training. Transition in care will be considered complete when ED triage or bedside nurses have assumed full care of the patient and EMS practitioners are no longer considered to be responsible for patient care.

#### Comparators

As this review is concerned with identifying transitional facilitators and barriers, we will include any comparator group or studies with no comparator.

#### Outcomes

We will include studies that examine the (1) experience and perceptions of transitions in care; (2) factors that mitigate or improve transitions in care; (3) quality of information exchanged between EMS practitioners and ED nurses; or (4) patient-level outcomes (clinical, satisfaction, patient-reported outcomes), provider- and team-level outcomes (satisfaction, time at triage, communication, teamwork), or system-level outcomes (medical errors, patient safety, quality of care, patient flow, costs) that are influenced by transitions in care. We will also report on any secondary outcomes described in the studies included in this review.

#### Study type

All English-language quantitative, qualitative, and mixed methods primary studies will be included, without restriction by year, publication type, geographic location, or methodological quality. Systematic reviews and other knowledge syntheses, case studies, editorials, and discussions of transitions of care will be excluded.

### Information sources and search strategy

We will search DARE, MEDLINE, EMBASE, Cochrane Central Register of Controlled Trials, CINAHL, and Joanna Briggs Institute EBP electronic databases from database inception for English-language publications. Gray literature will be searched for in databases (Proquest Dissertation and Theses Global, Web of Science Conference Proceedings Citation Index, OAIster, OpenGrey, Canadian Health Research Collection), relevant organizational websites (e.g., National Emergency Nurses Association, Paramedic Association of Canada, National Association of EMS Physicians), and consultations with clinicians. We will visually search the electronically available table of contents of key emergency nursing and EMS journals that are not indexed in two of the identified databases. The search strategy was developed by an information scientist (KAH). Table 1 provides the provisional search strategy for MEDLINE. We will search the reference lists and cited bys of included studies and will provide our final list of included studies to EMS and nursing experts in emergency medicine to identify any further studies for inclusions. Records will be exported to EndNote X8 to facilitate data management, removal of duplicates, and finding full texts.

**Table 1:** Provisional search strategy. Database(s): Ovid MEDLINE(R) Epub Ahead of Print, In-Process and other non-indexed citations, Ovid MEDLINE(R) Daily, and Ovid MEDLINE(R) 1946 to Present

#	Searches	Results
1	exp Patient Handoff/	691
2	exp Patient Transfer/	7049
3	exp Transfer Agreement/	253
4	hand-off*.mp.	375
5	handoff*.mp.	1388
6	hand-over*.mp.	921
7	handover*.mp.	1063
8	sign-over*.mp.	68
9	signover*.mp.	0
10	signout*.mp.	49
11	sign-out*.mp.	370
12	(transfer* adj care).mp.	95
13	(transition* adj care*).mp.	1231
14	(patient* adj transition*).mp.	622
15	(patient* adj transfer*).mp.	8814
16	(patient care adj report*).mp.	94
17	(triage adj report*).mp.	6
18	or/1–17	14,343
19	exp Emergencies/	38,186
20	exp Emergency Medical Services/	116,639
21	exp Emergency Service, Hospital/	62,927
22	exp Triage/	9682
23	exp Ambulances/	7791
24	(emergency adj2 room*).mp.	16,146
25	(emergency adj2 department*).mp.	70,119
26	(emergency adj2 unit*).mp.	3102
27	(emergency adj2 ward*).mp.	1218
28	“accident and emergency”.mp.	4460
29	“accident & emergency”.mp.	623
30	pre-hospital.mp.	3473
31	prehospital.mp.	9904
32	emergency medical service*.mp.	41,665
33	ambulance*.mp.	13,164
34	(triage adj2 room*).mp.	71
35	(triage adj2 department*).mp.	323
36	(triage adj2 unit*).mp.	79
37	(triage adj2 ward*).mp.	9
38	ems.mp.	10,144
39	paramedic*.mp.	7076
40	(emergency adj3 technician*).mp.	5893
41	(emergency adj3 responder*).mp.	837
42	(emergency adj3 personnel).mp.	1271
43	(emergency adj3 worker*).mp.	443
44	(emergency adj3 provider*).mp.	1114
45	(ems adj3 technician*).mp.	11
46	(ems adj3 responder*).mp.	49
47	(ems adj3 personnel*).mp.	565
48	(ems adj3 worker*).mp.	76
49	(ems adj3 provider*).mp.	691
50	or/19–49	217,243
51	18 and 50	3654
52	limit 51 to english language	3452

### Study selection

Prior to screening, we will conduct training and a calibration exercise with the review team to pilot and refine the screening tool in Excel. Titles/abstracts (level 1) will be independently screened by pairs of two reviewers. Disagreements will be resolved by a third reviewer. Full texts of potential studies (Level 2) will then be obtained and screening will be conducted in the same manner as level 1 screening.

### Assessment of methodological quality/risk of bias in individual studies

The methodological quality of quantitative studies will be assessed using the Effective Public Health Practice Project Quality Assessment Tool [EPHPP; 29], which can be used to assess multiple study designs and has evidence of validity and reliability. [27] Each of six domains—selection bias, study design, confounders, blinding, data collection methods, and withdrawals and drop-outs—are rated as *strong*, *moderate*, *weak*, or *not applicable*. For qualitative studies, we will use the Joanna Briggs Institute Critical Appraisal Checklist for Qualitative Research. [28] This coherent tool performs well in assessing intrinsic methodological quality. [29] Ten domains are assessed as *yes*, *not*, *unclear*, or *not applicable*: philosophy, objective, data collection, data analysis, interpretation of results, theory or cultural location, researcher reflexivity, participant representation, ethical considerations, conclusion. For mixed methods studies, we will use both appraisal tools. All studies will be independently assessed for quality by two reviewers, and disagreements will be resolved through discussion; if consensus cannot be reached, a third reviewer will break the tie. While we will report on the results of this quality assessment, studies will not be excluded on the basis of quality alone.

To assess confidence in the evidence in the syntheses, we will use the GRADE [30] (each quantitative outcome) and CERQual [31] (each qualitative review finding). GRADE and CERQual findings will be presented in a summary of findings table.

### Data items and data extraction

Table 2 details the data items to be extracted from the full texts. We will use a template data extraction tool in Excel. After the review team conducts a calibration exercise with the extraction tool, one reviewer will extract study data; a second reviewer will verify the extracted data for accuracy. We will discuss discrepancies and consult a third reviewer if a discrepancy cannot be resolved through consensus. Studies that have been published in duplicate will be retained and assessed in full text; we will combine related papers from the same study and treat them as one study.

**Table 2:** Data extraction categories for eligible studies

Category	Data extracted
Study characteristics	First author, year, country of origin, funding source, study objective, study design
Participants	Recruitment strategy, inclusion criteria, number of participants, demographic characteristics, professional designations
Setting and handover	Setting and context, descriptions of handover and relationships, tools to improve transitions in care
Intervention characteristics	Content, delivery method, unit of delivery, deliverer, setting, exposure, time, adherence
Outcomes and tools	Experience and perceptions of transitions in care; factors (barriers and facilitators) that influence transitions in care; quality of information exchanged between EMS practitioners and ED nurses; recommendations or tools to improve transitions in care; patient-level outcomes (clinical, satisfaction, patient-reported outcomes), provider- and team-level outcomes (satisfaction, time at triage, communication, teamwork), system-level outcomes (errors, patient safety, quality of care, patient flow, costs)

### Synthesis of included studies

Should the quantitative data permit, a meta-analysis will be conducted using a random effects model in RevMan (standardized mean differences for continuous outcomes; odds ratios for categorical outcomes; 95% confidence intervals). We expect considerable heterogeneity between studies; thus, meta-analysis may not be appropriate. In this case, we will aggregate the quantitative findings with the qualitative findings using data-based convergent synthesis [24], whereby one synthesis will be conducted with all studies after we transform the quantitative findings into textual categories or themes. Study characteristics will be tabulated (frequency, proportion), and narrative synthesis [32] will be used to integrate and explore relationships within the data. Where studies have substantial conceptual overlap, we will apply thematic analysis [33] in three stages to translate and integrate findings. Studies will be imported into NVivo 11 for coding. [34] First, two reviewers will inductively code first-order (verbatim participant accounts) and second-order interpretations (author observations) from the extracted data. Next, the initial codes will be compared between studies and grouped to generate initial descriptive themes. Finally, we will generate analytical themes by considering how the descriptive themes relate. Revisions to the coding framework will be recorded to provide an audit trail.

### Subgroups and sensitivity considerations

Providing that there is sufficient information, we will consider comparisons of several subgroups: (1) study types, (2) paramedics and nurses, (3) different countries, (4) transitions in care at triage and bedside, and (5) barriers and facilitators. We will also conduct a sensitivity analysis to examine the influence of studies with a low quality rating on the robustness of review findings. [35, 36] To do this, our synthesis (with all studies) will be compared post hoc to a synthesis without the methodologically weak studies within NVivo 11 using source classifications/attributes. The criteria or threshold for *low quality* (e.g., data collection method, sampling) will be established a priori. This comparison can provide insight into whether the low quality studies contribute unique information and how they may impact the generalizability of the findings. [35]

### Integrated knowledge translation

We are using an integrated knowledge translation [37] approach for this review. Our multidisciplinary team includes representative knowledge users who are frontline healthcare professionals, health system decision-makers, knowledge synthesis methodologist, information scientist, researchers (nursing, emergency medicine, EMS, health science), and trainees. Our team co-developed the review question and protocol and will continue to be involved throughout the entire process, including developing a knowledge translation plan to accelerate the uptake of the findings of the review. This strategy will detail the audiences, strategies with anticipated timing, key messages and activities, targeted venues, and measures of success. The findings of this review will be disseminated through multiple mechanisms: (1) open access publication; (2) presentations at targeted scientific conferences (e.g., Western Emergency Department Operations Conference); (3) presentations to provincial decision-makers and healthcare professionals through the Emergency Strategic Clinical Network; (4) meetings with EMS, manager, and nurse educators in local EDs; and (5) infographic representing meaningful information about transition in care. If indicated by the findings, this review will also inform the development of a communication transition tool to facilitate transfer of essential patient information in a consistent manner.

## Discussion

The findings from this systematic review will result in an in-depth understanding of the factors and interventional strategies that lead to improved communication and decrease in adverse events. To date, although EMS and ED nurses generally exhibit good working relationships, there is also a level of mistrust and sense of not being heard [1, 2]. Our review findings can inform the potential design and development of an evidence-informed transitions in care tool that ensures efficient transfer of accurate patient information, continuity of care, enhances patient safety, and avoids duplication of services. A standardized research-based approach to transitions in care would ensure that there are clear expectations of what information should be communicated and how. Given that ED triage nurses control access to the ED and are responsible for assigning priority to be examined and treatment space to arriving patients [15], efficient and accurate transitions in care at the initial point of contact with the ED can improve flow and optimize the use of ED resources. Ultimately, a standardized approach to transitions in care ensures that patient expectations and needs are communicated, thereby making ED care more responsive to patient needs.

The potential heterogeneity of the literature including varying study designs, study quality, and outcomes reported may constrain our ability to draw consistent and clear conclusions on the outcomes. Regardless of study heterogeneity, this systematic review will provide a meaningful analysis regarding the existing evidence on transitions in care between EMS and ED nurses and identify areas for future research.

## Abbreviations

CERQual: Confidence in the Evidence from Reviews of Qualitative Research
ED: Emergency Department
EMS: Emergency Medical Services
EPHPP: Effective Public Health Practice Project Quality Assessment Tool
GRADE: Grading of Recommendations Assessment, Development and Evaluation
